# Shearwaters sometimes take long homing detours when denied natural outward journey information

**DOI:** 10.1098/rsbl.2021.0503

**Published:** 2022-02-09

**Authors:** Oliver Padget, Natasha Gillies, Martyna Syposz, Emma Lockley, Tim Guilford

**Affiliations:** ^1^ Zoology Department, University of Oxford, Oxford, Oxfordshire, UK; ^2^ Queen Mary University of London, London, UK

**Keywords:** animal cognition, avian navigation, seabird, GPS tracking, seabird navigation

## Abstract

The cognitive processes (learning and processing of information) underpinning the long-distance navigation of birds are poorly understood. Here, we used the homing motivation of the Manx shearwater to investigate navigational decision making in a wild bird by displacing them 294 km to the far side of a large island (the island of Ireland). Since shearwaters are reluctant to fly over land, the island blocked the direct route home, forcing a navigational decision. Further still, on the far side of the obstacle, we chose a release site where the use of local knowledge could facilitate a 20% improvement in route efficiency if shearwaters were able to anticipate and avoid a large inlet giving the appearance of open water in the home direction. We found that no shearwater took the most efficient initial route home, but instead oriented in the home direction (even once the obstacle became visible). Upon reaching the obstacle, four shearwaters subsequently circumnavigated the land mass via the long route, travelling a further 900 km as a result. Hence, despite readily orienting homewards immediately after displacement, shearwaters seem unaware of the scale of the obstacle formed by a large land mass despite this being a prominent feature of their regular foraging environment.

## 1. Introduction

Manx shearwaters, *Puffinus puffinus*, and other procellariform seabirds do not routinely travel large distances over land [1]. In this system, topographic features, such as islands and peninsulas, create natural barriers in the environment that present contrasts between the shortest flyable route (over only water) and the direction home notwithstanding the obstacle (henceforth beeline, [2,3]). If homing is guided by true navigation (TN), navigation from beyond sensory contact with a goal and without the requirement for experience from that specific location using exocentric sensory cues [4,5], then this might be indicated when birds are unable to use knowledge of available routes that are more efficient than the beeline home. By contrast, the use of a familiar area map (a very general term for navigation based on experience, [6]) learnt over time, as better routes home from known sites are discovered and reinforced by the reward of arriving home efficiently [7], should result in birds being able to take routes that are more efficient than the beeline, when these exist.

When homing after foraging, free-ranging Manx shearwaters, on average, orient initially along with the beeline even from places where the beeline route is blocked by an island or peninsula, suggesting they use TN to encode a direction home without local route knowledge [2,3]. Several important features of this long-range navigation system remain unknown, however. First, how shearwaters would deal with intervening obstacles after artificial translocation, when relevant outward journey information (OJI) is unavailable. While OJI is known not to be crucial for successful homing in seabirds [8], it could facilitate homing in several ways. While path integration akin to that observed in *Cataglyphis* ants [9] is unlikely over such large distances, less precise course reversal (integration of the average, allocentrically derived compass bearing of the outbound route, [10,11]) could provide shearwaters with a homeward direction apparently blind to unflyable obstacles. Alternatively, if shearwaters remembered a sequence of landmarks during the outward journey, they could in theory reverse their outward route (route reversal, [12]). Second, it is unknown whether, in fact, local route knowledge can be learnt to improve efficiency, but only at locations from which the cost of failing to take an efficient route becomes large. If this were the case, it might indicate that birds are capable of forming a flexible familiar area map, but that to do so wholesale is prevented by cognitive or opportunist costs. Here, we displaced Manx shearwaters to a location within the likely normal foraging range of the population, where the anticipation of an unseen intervening island would result in an approximately 20% improvement in homing efficiency. This allowed us to observe how shearwaters behaved in response to an intervening obstacle (i) without natural OJI and (ii) when the cost of the difference between the beeline (predicted only by TN) route and the shortest path over only water (the most likely route given a familiar area map) was exaggerated.

## 2. Material and methods

**Figure 1.**
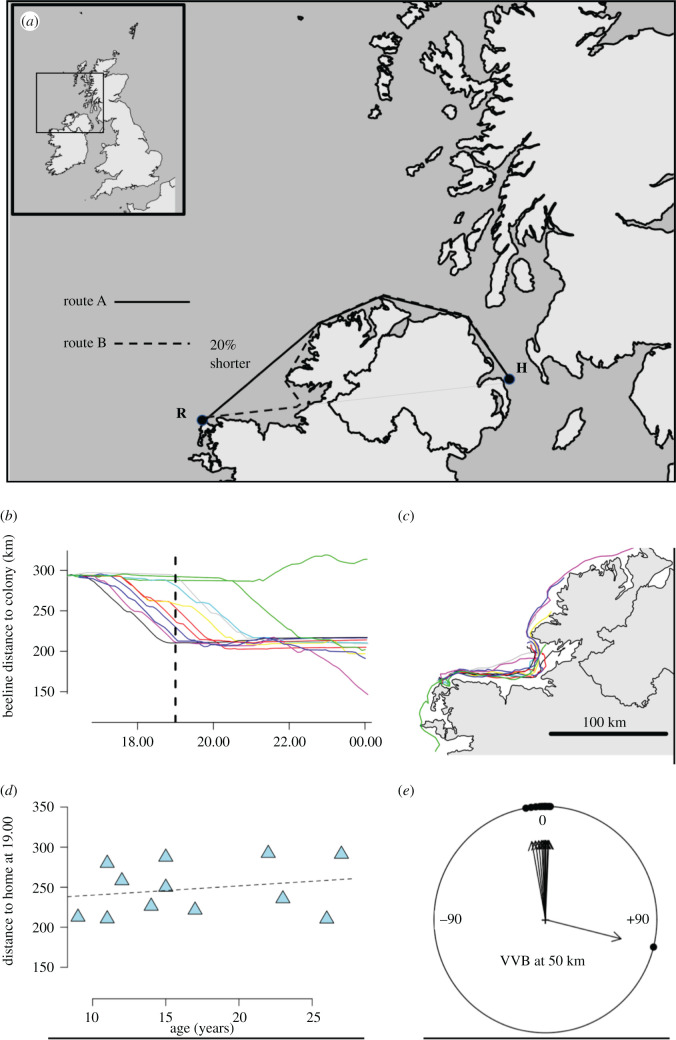
(*a*) Map showing the site of Lighthouse Island, Copeland (home,H) and the release site at Glenlara (R) with two hypothesized routes drawn. Route A is that expected if birds use locale-based familiarity to remember the most efficient route home. Route B is that expected by birds if they use TN. (*b*) The progress over the first day to the colony, measured as the beeline, Great Circle distance between the bird and the colony (the dashed line is at 19.00, the time at which the snapshot (*d*) is taken). (*c*) The tracks of released birds (*n* = 12) are shown also up until midnight on the day of release. (*d*) The relationship between bird age and progress to home at 19.00 on the day of release; (*e*) shows the virtual vanishing bearing (VVB) at 50 km (approximately 1 h travel for a shearwater, by which time the initial escape response should no longer be influencing the birds' navigational decisions), relative to the direction of the colony.

The study took place at Lighthouse Island, Copeland, Northern Ireland (54.695°N, 5.524°W). Incubation stints of Manx shearwaters from study nests (fitted with inspection hatches) were monitored prior to the experiment. On the morning of release (03/06/2019), we took birds of known age (between 9 and 27 years) from their nests that had recently taken over incubation stints but were not in the first day of the stint. During incubation, shearwaters are highly motivated to home [13–15], and this species exhibits excellent homing motivation when displaced after day 1 of an incubation stint [13]. While still at the colony, 17 g Mobile Action I-gotU gt-120 GPS loggers were attached dorsally using thin strips of tesa^®^ marine tape to small bunches of contour feathers. Devices were programmed to take fixes every 5 min (as in [16]). Birds were then transported from Lighthouse Island in towel-lined cardboard boxes, first to Donaghadee by boat and then to the release site by car. To maximally exaggerate the difference in cost between the routes with and without anticipation of the intervening obstacle, we chose to displace birds 294 km to the northwest-most tip of Co. Mayo (Glenlara, Republic of Ireland, 54.289°N, 9.988°W, R, figure 1*a*). From here, the most efficient route flying only over water would require northeast initial orientation to cut the corner to the north of the island of Ireland, a route of roughly 376 km (figure 1*a*). If birds were initially to orient along the beeline, failing to take the shortcut to the northern tip of Ireland, this distance increases to approximately 455 km since birds will reach the coast of the intervening island and then have to circumnavigate the topography to the north to reach home flying only over water. Shearwaters were released singly from 8 m elevation by tossing them out to sea from Glenlara, each only after the previous bird had vanished from sight.

The ‘virtual vanishing bearing’ (VVB), defined as the acute angle between the release site, a bird's location and initial goal, was measured with respect to the shortest route (route A, figure 1*a*) and the beeline to Lighthouse Island, Copeland (route B, figure 1*a*) when the birds reached 50 km distance from the release site, approximately halfway between the release site and the topographic obstacle. All analyses were done in R, and maps were produced with the ‘maps’ package [17,18].

## 3. Results

**Figure 2.**
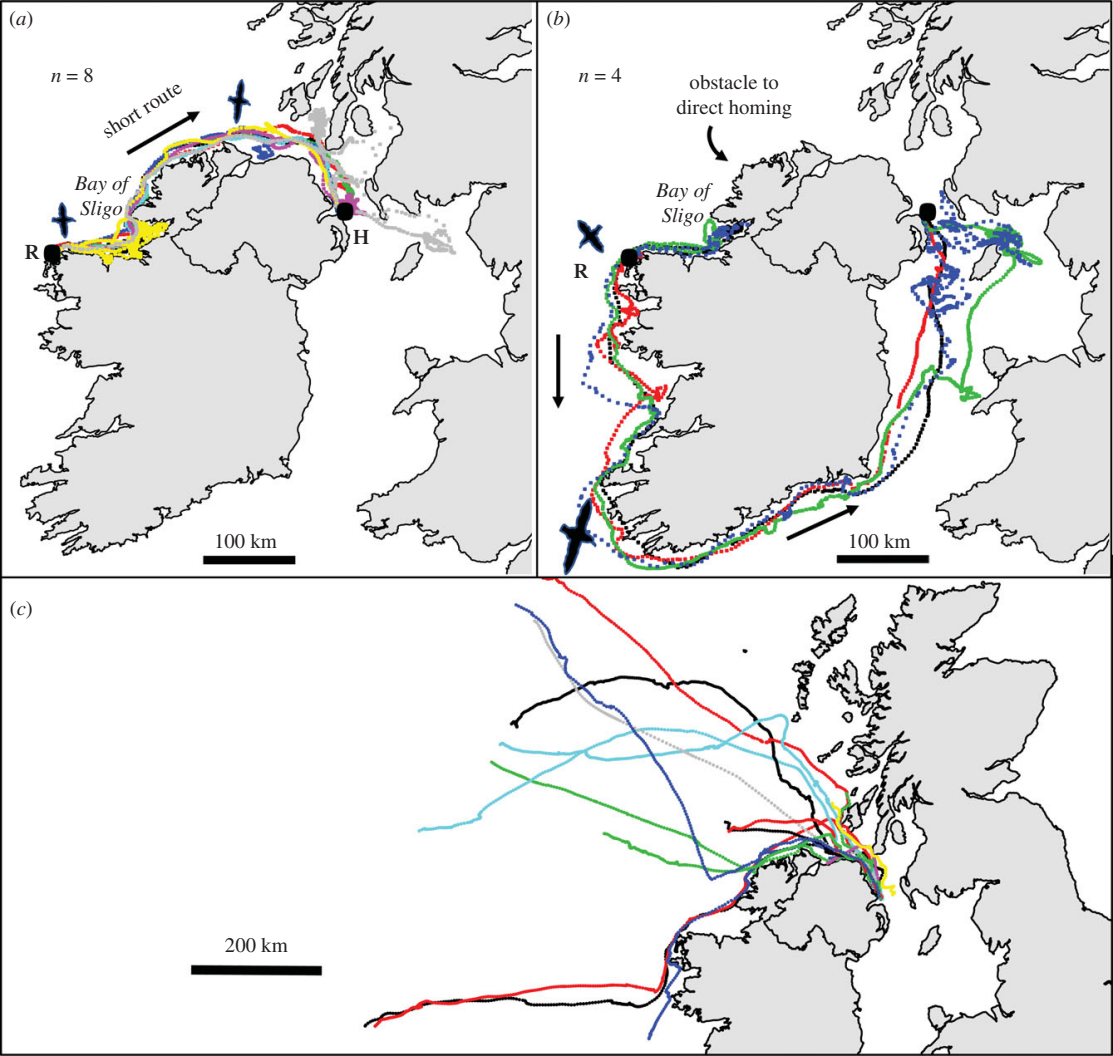
The tracks of released birds that go the short route (*a*) and long route (*b*) around the island of Ireland are shown; (*c*) shows the homing trips of free-ranging shearwaters (17 trips from 16 birds) hitting the coast of Ireland and proceeding via the short route on their homing journeys [2,3].

All 13 shearwaters were recovered at Copeland, between 2 and 9 days after displacement (see electronic supplementary material, table S1). Of these 13, 12 GPS loggers recorded full homing tracks. Eleven out of these 12 birds initially were oriented toward the colony, close to the less efficient beeline rather than the shortest flyable route, with a mean deflection from the beeline, at 50 km from release, of +3.5° (bootstrapped 95% CI [−3.49, 19.26]) in comparison with the mean deflection from the shortest path home of +36.3° (bootstrapped 95% CI [29.47, 51.64]). Once birds reached the Bay of Sligo, eight proceeded to home by following the coast around the north of Ireland. Three, however, reversed their initial movement from the release site and, similarly to the bird that did not initially display homeward orientation at the release site, followed the coast around the island of Ireland in the opposite direction, travelling 910 km further as a result (figure 1*b,c*). In this small sample, there was no detectable effect of bird age on the time that birds took to cover the first 50 km towards home (linear regression: *t*_10_ = 0.705, *p* = 0.497, figure 1*d*), or in their propensity to proceed to home via the short or long coastal routes (logistic regression: *z* = 1.020, *p* = 0.308, figure 1*e*). The tracks of all displaced birds are shown in figure 2*a,b*.

## 4. Discussion

While long-distance displacement experiments with pigeons [19] were crucial in revealing the sensory cues required by some birds for homing from beyond their familiar area, they are more limited in revealing the mechanisms underpinning the natural navigation of wild birds that are seldom in entirely novel places or that lack information from the outward journey [2,3,7]. Here, rather than controlling previous experience, we observed decisions made during navigation in a wild seabird, from which we inferred whether local route knowledge was being used. We found that without natural OJI, shearwaters exhibited excellent initial homing orientation toward the colony, but were unable to anticipate an unflyable obstacle, or avoid long detours by choosing the shorter route around that obstacle when their path became blocked.

Free-ranging shearwaters have knowledge of both direction and distance home, inferred from their correctly timed homing journeys [2,3,20–22]. As with the displaced birds presented here, initial orientation in Manx shearwaters appears not to be informed by previous experience of homing from those locations, since in both circumstances shearwaters apparently fail to anticipate large obstructions to the homing path. In the current study, passive displacement of birds additionally precludes the use of OJI for course reversal [9,11] or route reversal [12]. Rather, homing is probably facilitated by TN, since this is the only mechanism that provides birds with a homing vector not contingent on local route knowledge. TN is normally considered to involve consulting a large-scale map based on gradients of allocentric environmental cues (atmospheric odours and magnetic cues are the most seriously entertained candidates for birds, [14,23]). So while birds could, hypothetically, use a TN map to execute routes other than the beeline (for instance, if the location of an intermediate goal was encoded in a TN map by its cue coordinates), adherence to the beeline is nonetheless diagnostic of a navigational mechanism that encodes a homeward direction via a large-scale, generalized ‘map’ that does not incorporate local route knowledge. It is prudent to acknowledge that this inference is contingent on the assumption that there is not a hidden benefit for apparently taking the beeline, such as an aerodynamic advantage to travelling along the coast. We think this is especially unlikely, however, (i) because, from the chosen release site, the potential improvement in overall route distance was large (20%); (ii) because as homing progressed that percentage increased considerably as the birds reached the Bay of Sligo (figure 2*a,b*); and (iii) because free-ranging birds homing naturally from foraging trips cut across the Bay of Sligo rather than closely follow the coast, implying that here shearwaters do not naturally seek any benefit of coastal travel (figure 2*c*).

After reaching the Bay of Sligo, displaced shearwaters often (4/12 tracked birds) failed to home via the shorter route around the island of Ireland, despite a 900 km difference in distance between the two route options (figure 2*a,b*). This failure to take the shorter coastal route contrasts distinctly with the natural, free-ranging homing behaviour observed in previous GPS tracking studies of Manx shearwaters from the same colony. A crude *post hoc* comparison between the homing trajectories of displaced birds presented here and the 17 free-ranging birds from the published data of Padget *et al*. [3], where shearwaters from Copeland flew beyond the island of Ireland into the northeast Atlantic (figure 2*a–c*), suggests that removal of relevant OJI had an impact on birds' ability to make navigational decisions (Fisher's exact test comparing 4/12 with 0/17: *p* < 0.05). Taking only free-ranging shearwaters, one possibility is that shearwaters are familiar with the coast and hence able to determine the shortest route from experience when an intervening island is reached but that familiar coastlines are not nested in the same spatial representation as the TN map that provides initial homeward orientation. However, this does not account easily for why displaced birds were unable to achieve the same. We can think of three hypotheses for this disparity. **Hypothesis 1:** If free-ranging birds tend to repeat foraging journeys, then our natural homing subset may be more familiar with homing from beyond Ireland than those birds chosen for displacement, leading to a sampling artefact. **Hypothesis 2:** Displaced birds are familiar with homing from beyond Ireland, but flight from the release site and along the route home after displacement did not bring shearwaters to a location from which they recognized the view of the coast. On the other hand, free-ranging shearwaters are able to simplify route recognition by travelling towards a coast along a stereotyped trajectory and can therefore make use of their experience to circumnavigate Ireland via the shorter route. The involvement of landmark recognition with familiar area route following has been discussed with respect to homing pigeon navigation [6] and has parallels with active vision, where stereotyped approaches improve object recognition in insects and chickens [24]. **Hypothesis 3:** Shearwaters make use of some, sparse information collected during the outward journey: ‘auxiliary outward journey information’. For example, if birds remember the predominant side that land appeared on the outward journey and then effect a turn in the same direction (from an egocentric point of view) when they later encounter an obstacle, this would on average lead them to the open water route taken on the outward journey. This relatively simple mechanism, or something similar, could facilitate homing challenges requiring detours while demanding little cognitive complexity.

Our findings show that, while TN appears to provide shearwaters with the direction home even after long and tortuous natural foraging trips, it is not, on its own, sufficient to avoid poor route efficiency following displacement. Interestingly, shearwaters appear to have behaviours that allow them to cope with this, suggested by the observation that shearwaters thwarted in their choice of the beeline are clearly then able to switch to a navigational plan B involving coast following and, ultimately, successfully home to the colony. For four birds, this involved flying for more than 500 km in a direction away from home, illustrating considerable flexibility in solving navigational tasks. That free-ranging shearwaters avoid this problem provides early validity to the intriguing idea that wild navigators use TN in combination with additional relevant information collected during the outward journey on natural foraging trips, illustrating the need for a diversity of approaches to understanding navigation based on both displacement experiments and the careful analysis of free-ranging movement. Future displacements to sites where the likely familiarity of the release site and the costs of incorrect route discrimination are varied might disentangle the precise mechanisms that supplement TN in wild birds, since release site factors relate to a shearwater's opportunity to learn an efficient route and the potential efficiency yield from learning it.

## Data accessibility

GPS tracking data are deposited with Dryad and available to download from: https://doi.org/10.5061/dryad.8pk0p2npp [25].
